# Supplementary material for: Coumarins and Hesperetin Inhibit Human Respiratory Syncytial Virus Infection
Source: Int J Mol Sci. 2024 Dec 11;25(24):13301. doi: 10.3390/ijms252413301 (PMC11676883; doi:10.3390/ijms252413301)
Supplement: Supplementary file 1 [file ijms-25-13301-s001.zip › ijms-3311027-supplementary.pdf]

Supplementary Materials

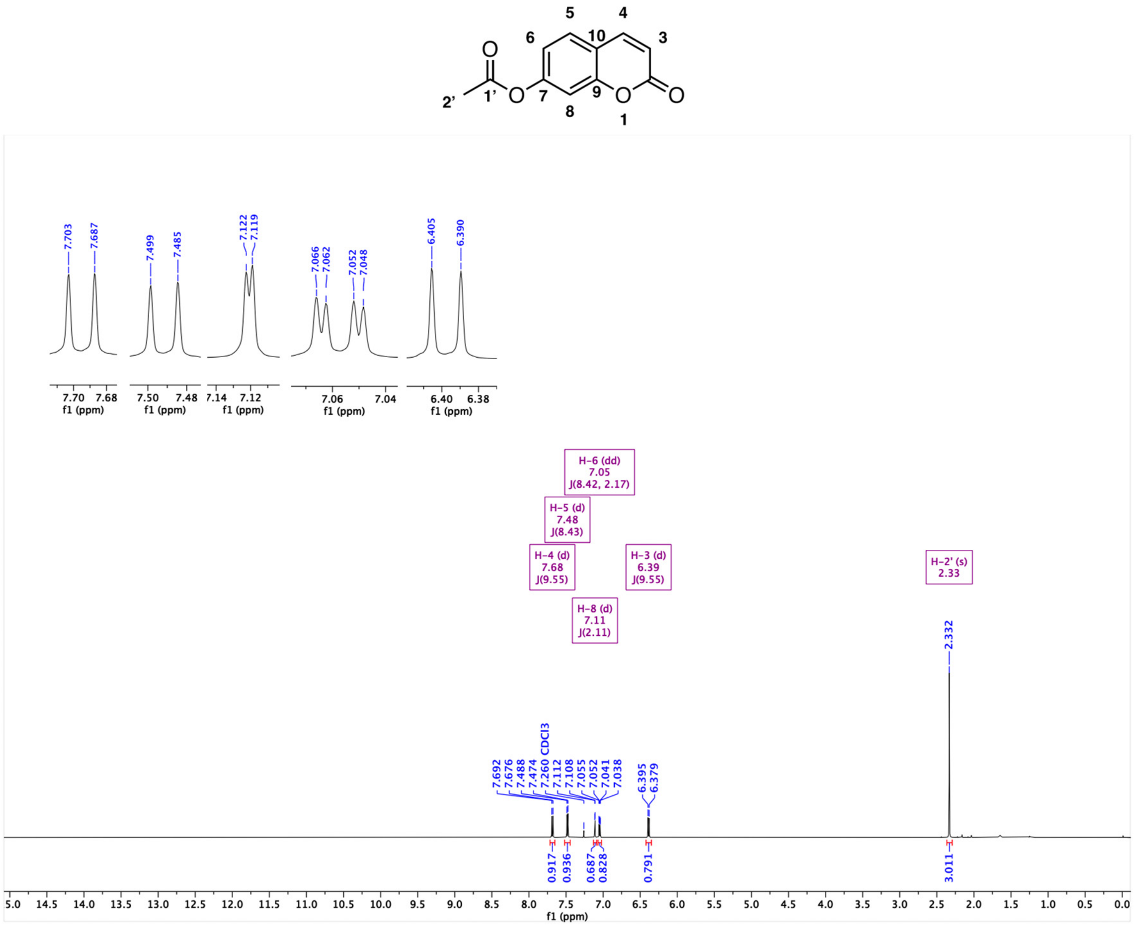

Figure S1: ^1H NMR spectrum of 7-acetoxycoumarin ($CDCl_3$; 600 MHz)

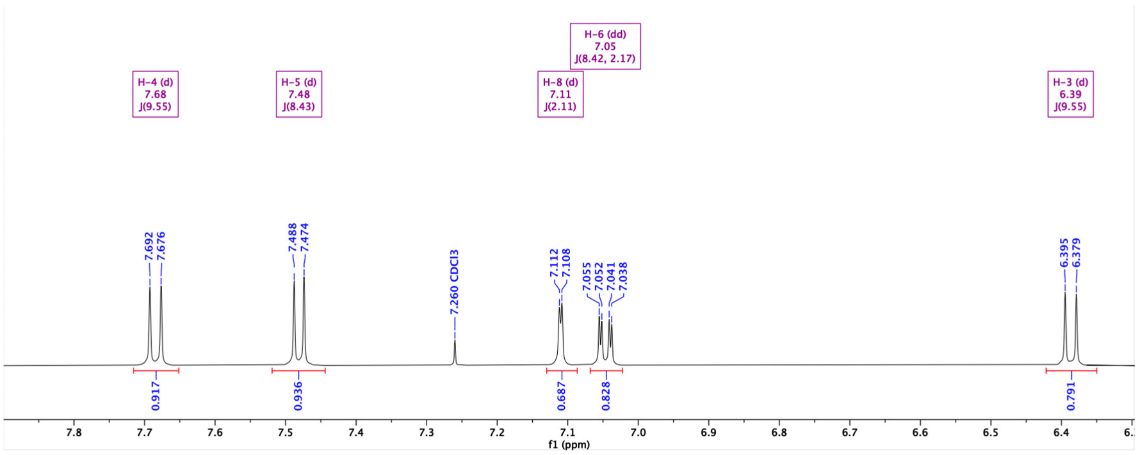

Figure S2: Amplification of ^1H NMR spectrum of 7-acetoxycoumarin (6.4 – 7.9 ppm) ($CDCl_3$; 600 MHz)

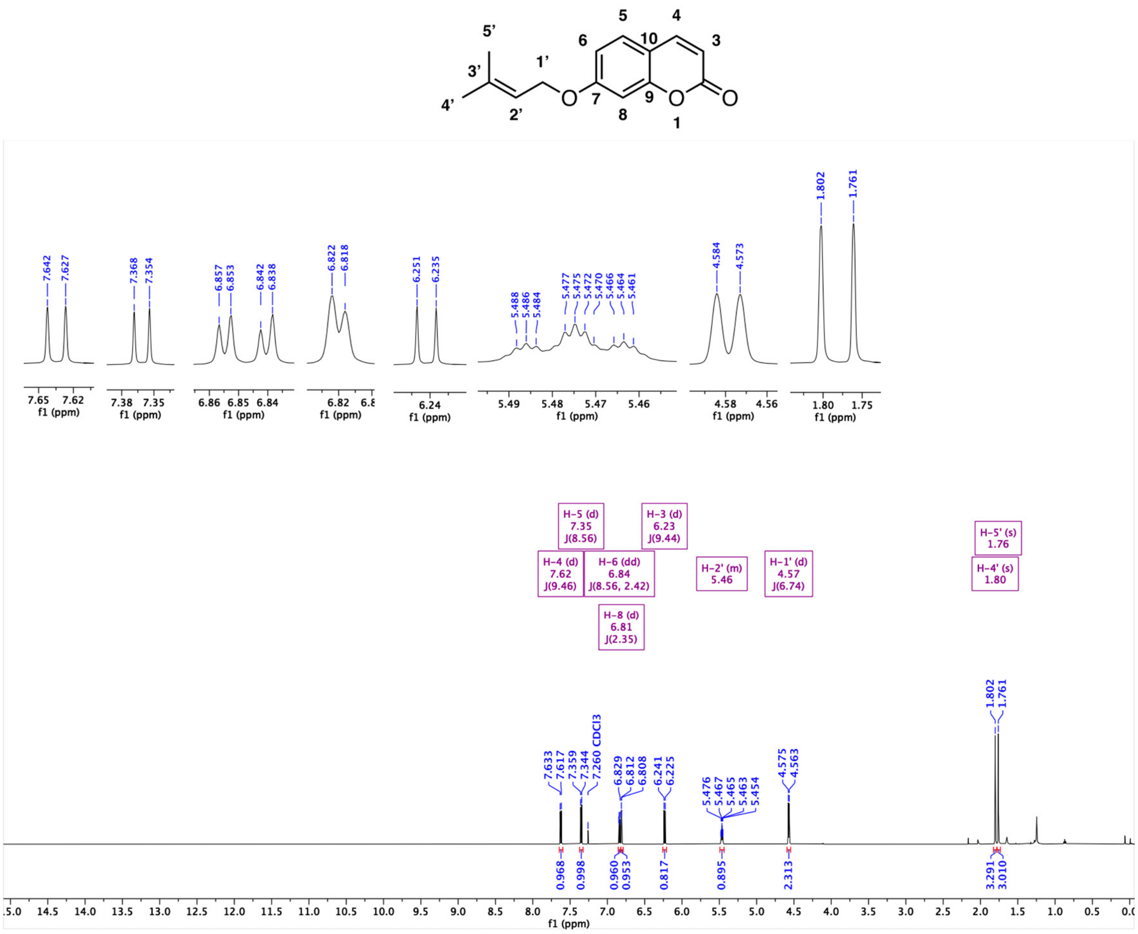

Figure S3: ^{1}H NMR spectrum of 7-isopentenyloxycoumarin ($CDCl_3$; 600 MHz)

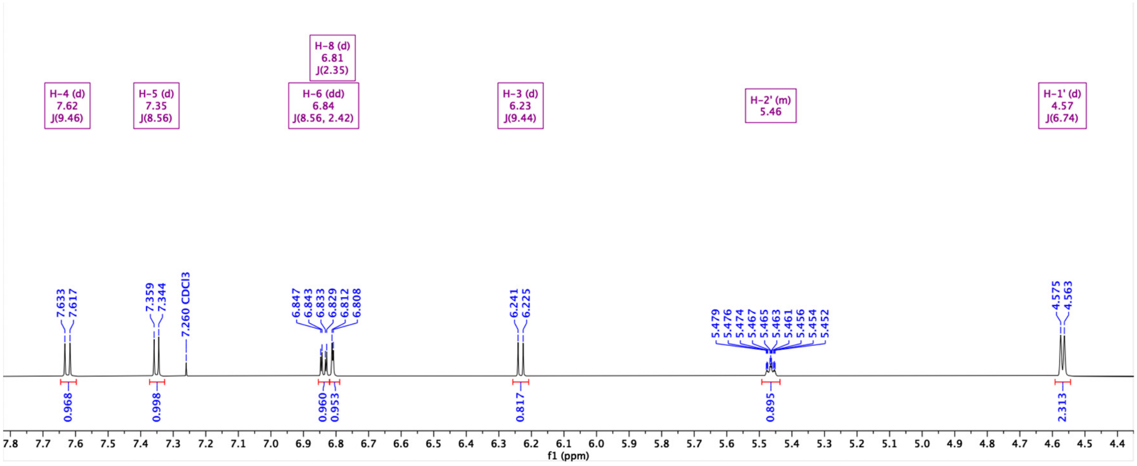

Figure S4: Amplification of ^{1}H NMR spectrum of 7-isopentenyloxycoumarin (4.4 – 7.8 ppm) ($CDCl_3$; 600 MHz)

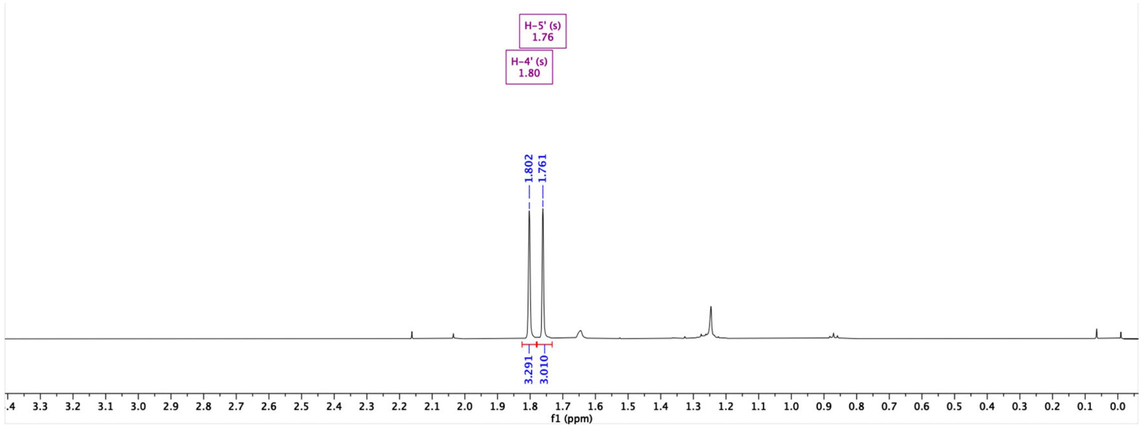

Figure S5: Amplification of ^{1}H NMR spectrum of 7-isopentenyloxycoumarin (0.0 – 3.4 ppm) ($CDCl_3$; 600 MHz)

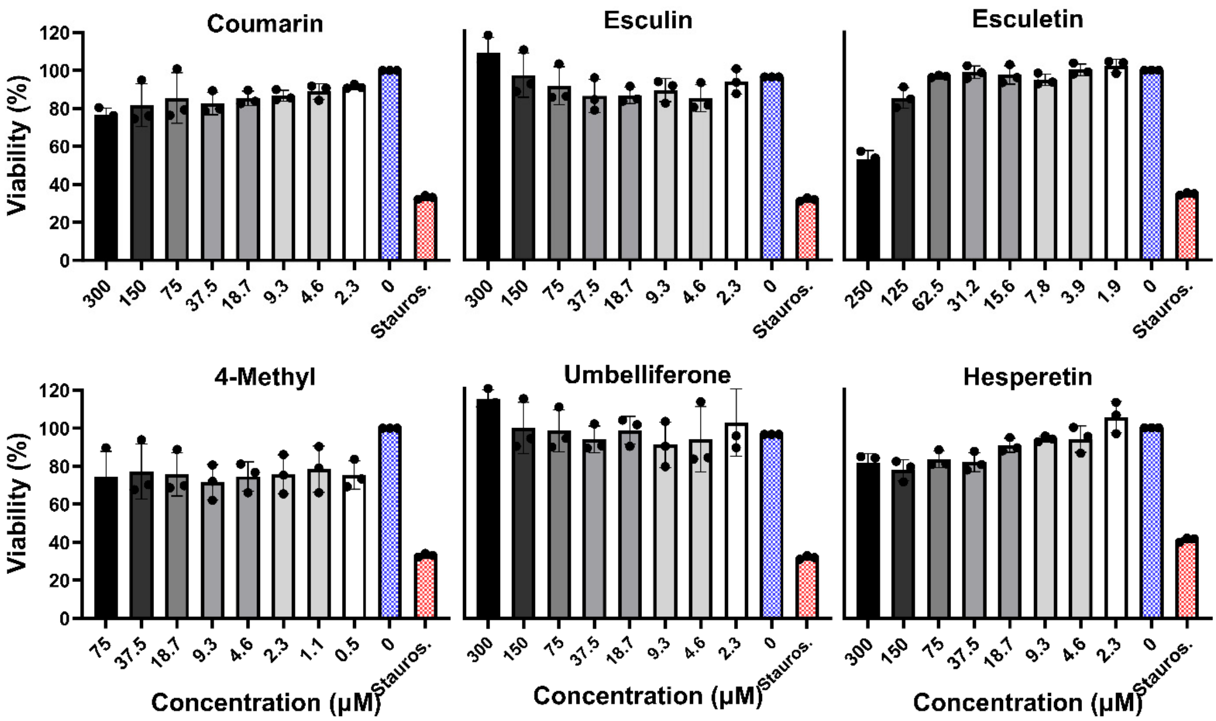

Figure S6: Cytotoxicity assay of compound on A549 cells at 48 hours of incubation with the compounds: coumarin, esculetin, esculin, 4-methyl esculetin, umbelliferone and hesperetin. The blue bar represents positive control (untreated) and red bar represents Staurosporine (1 mM), negative control, in which 50% of cells are unavailable.

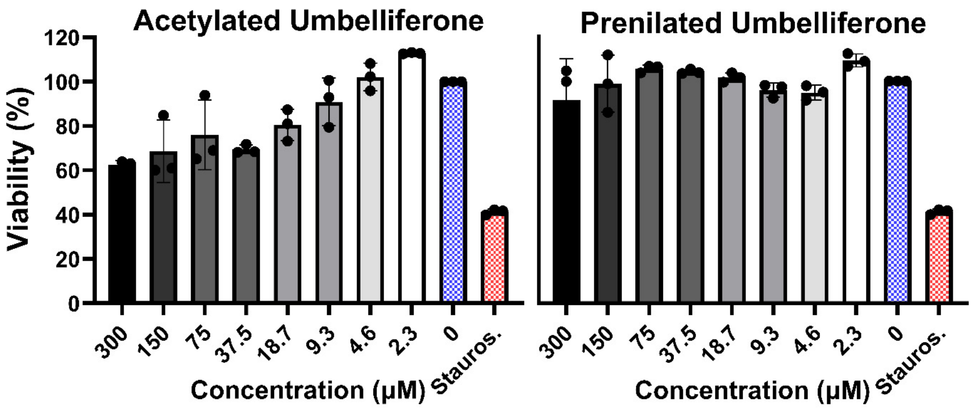

Figure S7: Cytotoxicity assay on A549 cells at 24 hours to acetylated and prenylated umbelliferone. The blue bar represents the positive control which had only cells and the red bar represents the negative control (Staurosporine).

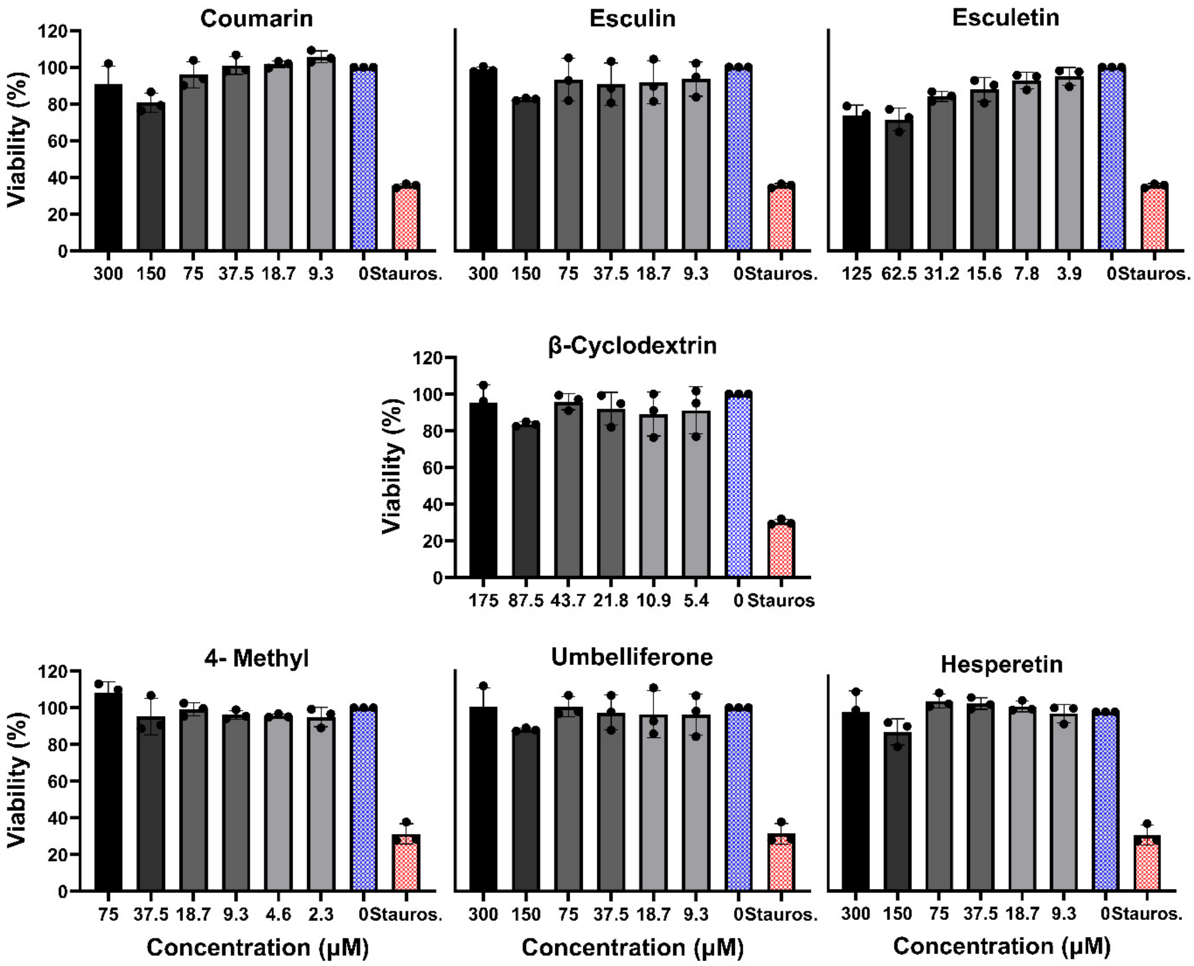

Figure S8: Cytotoxicity assay on A549 cells of compounds encapsulated with β- Cyclodextrin at 48 h. The blue bar represents the positive control which had only cells and the red bar represents the negative control (Staurosporine).
